# Enzalutamid versus aktive Überwachung bei Patienten mit Low-risk-Prostatakarzinom

**DOI:** 10.1007/s00066-022-02015-z

**Published:** 2022-10-19

**Authors:** Jürgen Dunst, David Krug

**Affiliations:** Klinik für Strahlentherapie Kiel, UKSH, Kiel, Deutschland

## Hintergrund

Für Patienten mit Low-risk-Prostatakarzinom gelten aktive Überwachung, Strahlentherapie und Prostatektomie als anerkannte Therapieoptionen. Eine alleinige medikamentöse antihormonelle Therapie ist primär nicht indiziert und wird in der S3-Leitlinie nur empfohlen, wenn entweder Therapiebedarf besteht und die Standardtherapieverfahren nicht möglich sind oder wenn eine kurative Therapie abgelehnt wird [8]. In der Leitlinie wird explizit gefordert, Patienten über den palliativen Charakter dieser Option sowie die mit einer hormonablativen Therapie verbundenen unerwünschten Wirkungen und die uneinheitliche Datenlage bezüglich eines Überlebensvorteils aufzuklären. In den einschlägigen Metaanalysen gibt es nämlich keinen Überlebensvorteil durch eine primäre antihormonelle Therapie bei Patienten mit Low-risk-Prostatakarzinom, sondern nur bei Vorliegen erheblicher Risikofaktoren, insbesondere T3–4, PSA > 50 μg/l und PSA-Verdopplungszeit unter einem Jahr [1, 7]. In den vor etwa 20 Jahren durchgeführten Studien mit Bicalutamid wurde der Studienteil für Patienten mit aktiver Überwachung vorzeitig geschlossen [10]. Auch in neoadjuvanten Konzepten wurden keine relevanten Effekte erzielt [2, 6].

Der Androgenrezeptorinhibitor Enzalutamid gehört, zusammen mit Apalutamid, Darolutamid und Abirateron, zu den neuen, sehr wirksamen Substanzen beim Prostatakarzinom. Diese Substanzen wurden primär bei Patienten mit kastrationsrefraktärer Erkrankung in Kombination mit einer androgendeprivativen Therapie getestet und zugelassen. Enzalutamid ist mittlerweile auch in der First-line-Therapie des metastasierten hormonsensitiven Prostatakarzinoms zugelassen [5]. Der Versuch, das Indikationsspektrum auf die Primärtherapie der lokal begrenzten Tumoren zu erweitern, ist deshalb durchaus verständlich. In der ENACT-Studie, die dies untersuchte, sind die Ergebnisse aber – nicht unbedingt überraschend – eher enttäuschend [9].

## Patienten und Methodik

ENACT ist eine randomisierte Therapiestudie bei Patienten mit Low- bzw. Intermediate-risk-Prostatakarzinom (nach „NCCN guideline“); Patienten mit „very low risk“ (Gleason-Score 6, < 3 Stanzen pos., PSA < 10 μg/l) waren ausgeschlossen. Im Kontrollarm erfolgte eine aktive Überwachung (AS), im experimentellen Arm erhielten Patienten zusätzlich Enzalutamid 160 mg/d für ein Jahr. Kontrollbiopsien waren nach einem und 2 Jahren vorgesehen. Der primäre Endpunkt war die Zeit bis zur pathologischen (Progression in der Kontrollbiopsie mit entweder Gleason-Score-Zunahme oder Anstieg der positiven Stanzen um ≥ 15 %) oder therapeutischen Progression (Beginn einer spezifischen Therapie). Sekundäre Endpunkte waren die Rate negativer Biopsien nach einem bzw. 2 Jahren und die Zeit bis zur PSA-Progression (definiert als ein Anstieg um mindestens 2 μg/l oder 25 % über Nadir).

## Ergebnisse

Zwischen Juni 2016 und August 2020 wurden 227 Patienten rekrutiert (114 mit AS plus Enzalutamid, 113 mit AS allein), überwiegend mit Gleason-Score 6 (59 %), der Rest mit Gleason-Score 7a. Nach einem Jahr zeigte sich ein signifikanter Vorteil im Hinblick auf den primären Endpunkt; die Rate an pathologischen bzw. therapeutischen Progressionen betrug 23 % bei AS und 7,9 % bei AS plus Enzalutamid (Odds Ratio 0,3, *p* < 0,01). Aber bereits nach 2 Jahren war die Progressionsrate mit 16,4 % bzw. 16 % identisch. Ferner bestanden nach einem Jahr auch signifikante Vorteile in den sekundären Endpunkten PSA-Progression (HR 0,71, *p* = 0,03) und Rate negativer Kontrollbiopsien (Odds Ratio 3,5, *p* < 0,001), aber auch diese Vorteile waren nach 2 Jahren nicht mehr signifikant. Die Rate von unerwünschten Ereignissen war unter Enzalutamid signifikant erhöht (92,0 % versus 54,9 % im Kontrollarm). Im Enzalutamidarm berichteten 88,4 % der Männer über unerwünschte Nebenwirkungen im Zusammenhang mit dem Medikament; in 2,7 % wurden diese als schwerwiegend eingestuft, und in 7,1 % führten sie zum Abbruch der Enzalutamidtherapie.

## Schlussfolgerungen

Die Autoren folgern, dass Enzalutamid gut vertragen wurde, ein signifikantes Ansprechen bewirkte und daher eine mögliche Behandlungsoption für Patienten mit Indikation zur aktiven Überwachung darstellen könnte.

## Kommentar

Dass die Herstellerfirma zusammen mit klinischen Forschern diese Studie aufgelegt und durchgeführt hat, ist wissenschaftlich begründet und legitim. Die Interpretation der Ergebnisse ist aber absolut inakzeptabel. Die Arbeit wurde zudem durch einen Kommentar im selben Heft („invited commentary“) gewürdigt [3]; die Kommentatoren hinterfragen zwar einige Aspekte (v. a. den ungewöhnlichen primären Endpunkt, die Frage der möglichen Resistenzentwicklung mit daraus resultierender eingeschränkter Wirksamkeit bei Metastasierung und die hohen Kosten von 150.000 $ pro Jahr), aber auch sie sprechen von „encouraging data“ und verschweigen die wesentlichen Kritikpunkte:

- Am Ende der einjährigen Therapie mit Enzalutamid gab es zwar signifikante Effekte; diese waren aber bereits ein Jahr später verschwunden.
- 15–20 % der Patienten zeigten bereits unter der Enzalutamideinnahme eine PSA-Progression; diese Information steht nicht im Text, ergibt sich aber aus einer der Abbildungen.
- 15 Monate nach der Randomisierung (also bereits 3 Monate nach Ende der Enzalutamidgabe) kreuzen sich die Kurven der PSA-Rezidivfreiheit. Ein nachhaltiger Effekt sieht anders aus.
- Etwa 9 von 10 Männern berichteten über unerwünschte Ereignisse im Zusammenhang mit der Medikation; in 2,7 % waren diese vom Schweregrad 3 oder stärker. Diese Therapie als gut verträglich zu bezeichnen, ist aus unserer Sicht mindestens fragwürdig oder schlicht und einfach falsch. Daten zur Lebensqualität werden nicht erwähnt; man kann über den Grund spekulieren.
- Diese Therapie führt das Konzept der aktiven Überwachung, das ja Therapieverzicht bedeutet, ad absurdum.
- Die Kosten (in den USA offensichtlich 150.000 $ pro Jahr, in Deutschland ja aktuell etwas mehr als 40.000 € pro Jahr) sprechen für sich.
- Für einen Radioonkologen am schlimmsten ist aus unserer Sicht die Tatsache, dass sowohl in der Diskussion als auch im Kommentar eine andere Alternative zur aktiven Überwachung nicht erwähnt wird; die exzellenten Daten der modernen Strahlentherapie bezüglich Effektivität und Verträglichkeit werden verschwiegen. Die CEASAR-Studie hat nämlich kürzlich gezeigt, dass in diesem Patientenkollektiv die Strahlentherapie zu fast keinen funktionellen Einschränkungen im Vergleich zur aktiven Überwachung führt; ein solches Ergebnis ist praktisch nicht zu toppen [4]. Die Strahlentherapie ist eine sehr effektive und kostengünstige Behandlung. Damit kann sich eine medikamentöse Therapie nicht messen.

## Fazit

Eigentlich braucht man diese Studie nicht ernst nehmen, und das Medikament ist ja auch bisher für diese Indikation gar nicht zugelassen. Dennoch sollten wir bei jeder Gelegenheit deutlich machen, dass eine medikamentöse Therapie keine Alternative zur Strahlentherapie bei prognostisch günstigen Prostatakarzinomen ist.

*Jürgen Dunst & David Krug, Kiel*
